# Multi-Omics Analysis Reveals Myelin, Presynaptic and Nicotinate Alterations in the Hippocampus of G72/G30 Transgenic Mice

**DOI:** 10.3390/jpm12020244

**Published:** 2022-02-09

**Authors:** Michaela D. Filiou, Larysa Teplytska, Markus Nussbaumer, David-M. Otte, Andreas Zimmer, Christoph W. Turck

**Affiliations:** 1 Proteomics and Biomarkers, Max Planck Institute of Psychiatry, 80804 Munich, Germany; L.Teplizkaja@web.de; 2 Department of Biological Applications and Technology, School of Health Sciences, University of Ioannina, 45110 Ioannina, Greece; nussbaumermarkus.80@gmail.com; 3 Biomedical Research Ιnstitute, Foundation for Research and Technology-Hellas, 45110 Ioannina, Greece; 4 Institute of Molecular Psychiatry, University of Bonn, 53125 Bonn, Germany; d.otte@uni-bonn.de (D.-M.O.); a.zimmer@uni-bonn.de (A.Z.); 5 Center of Aptamer Research and Development, LIMES Institute, University of Bonn, 53121 Bonn, Germany

**Keywords:** G72, schizophrenia, proteomics, Sirt2, metabolomics, hippocampus, myelination, ^15^N metabolic labeling, mice, mitochondria

## Abstract

The primate-specific G72/G30 gene locus has been associated with major psychiatric disorders, such as schizophrenia and bipolar disorder. We have previously generated transgenic mice which carry the G72/G30 locus and express the longest G72 splice variant (LG72) protein encoded by this locus with schizophrenia-related symptoms. Here, we used a multi-omics approach, including quantitative proteomics and metabolomics to investigate molecular alterations in the hippocampus of G72/G30 transgenic (G72Tg) mice. Our proteomics analysis revealed decreased expression of myelin-related proteins and NAD-dependent protein deacetylase sirtuin-2 (Sirt2) as well as increased expression of the scaffolding presynaptic proteins bassoon (Bsn) and piccolo (Pclo) and the cytoskeletal protein plectin (Plec1) in G72Tg compared to wild-type (WT) mice. Metabolomics analysis indicated decreased levels of nicotinate in G72Tg compared to WT hippocampi. Decreased hippocampal protein expression for selected proteins, namely myelin oligodentrocyte glycoprotein (Mog), Cldn11 and myelin proteolipid protein (Plp), was confirmed with Western blot in a larger population of G72Tg and WT mice. The identified molecular pathway alterations shed light on the hippocampal function of LG72 protein in the context of neuropsychiatric phenotypes.

## 1. Introduction

The G72/G30 gene area on human chromosome 13q is a susceptibility locus for major psychiatric disorders, including schizophrenia and bipolar disorder [1,2]. The main protein product of this locus is the longest G72 splice variant (LG72) protein (G72 protein, also termed D-amino acid oxidase activator (DAOA)), whose function in relation to psychiatric disorders remains obscure [3]. Elevated G72 protein levels were found in the serum of patients suffering from schizophrenia [4] and a tendency towards increased expression of the G72 gene has been observed in brains of schizophrenia patients [5].

To study the function of the G72 protein in vivo, we have generated humanized transgenic mice carrying the G72/G30 locus (G72Tg) that express the G72 protein [6]. G72Tg mice exhibit schizophrenia-related symptoms, including impaired motor coordination, sensorimotor gating and olfactory discrimination, increased compulsive behavior as well as spatial memory deficits [6,7]. Intriguingly, treatment with the antipsychotic haloperidol was shown to reverse sensorimotor gating impairment in a prepulse inhibition paradigm in G72Tg mice [6]. At the molecular level, we have characterized the cerebellar profiles of G72Tg mice using complementary proteomics approaches, including quantitative mass spectrometry (MS) [8] and 2D-gel electrophoresis, and found protein expression alterations in myelin-, mitochondria- and oxidative stress-related processes in G72Tg compared to wild-type (WT) mice [7,9].

Here, our aim was to investigate G72-induced changes in the mouse hippocampus in order to shed light on the LG72 function in vivo and in relation to neuropsychiatric phenotypes. For this purpose, we implemented a multi-omics approach, based on quantitative proteomics and metabolomics in order to explore altered molecular biosignatures and networks in the hippocampi of G72Tg vs. WT mice. Selected protein changes identified by quantitative proteomics were then verified by Western blot analysis.

## 2. Materials and Methods

### 2.1. Experimental Design

**Figure 1:**
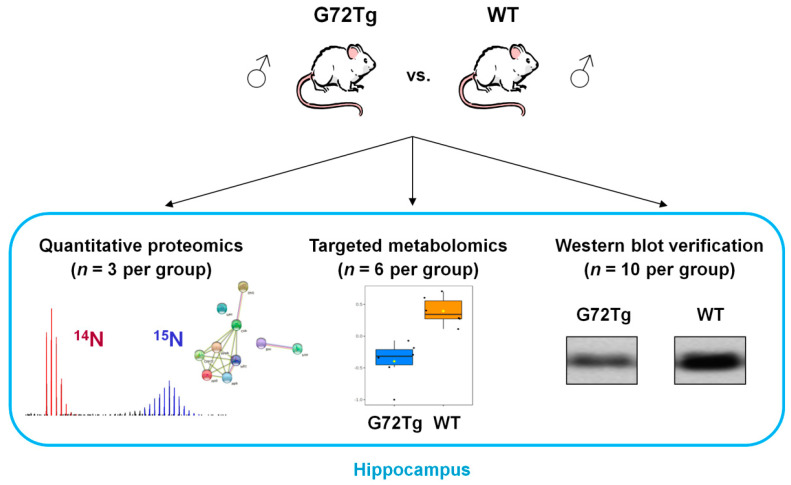
Experimental design. Transgenic mice carrying the G72/G30 locus (G72Tg) vs. wild-type (WT) male mice hippocampi were compared by ^15^N metabolic labeling-based quantitative proteomics and targeted metabolomics. Selected protein expression alterations were verified in a larger cohort of G72Tg and WT mice.

All mice studied were males, eight weeks old of CD1 background. For quantitative proteomics, we compared three hippocampi each of G72Tg and WT mice by quantitative MS, using ^15^N metabolically labeled hippocampi of CD1 mice as internal standards. For targeted metabolomics, we compared the hippocampi from six G72Tg and six WT mice. Ten G72Tg and 10 WT mice were used for verification of the identified proteomics results. The experimental design of the study is provided in Figure 1.

### 2.2. Transgenic Mice Carrying the G72/G30 Locus (G72Tg) and Wild-Type (WT) Mice

G72Tg mice were generated, bred and genotyped as described previously [6,7]. The G72Tg and WT mice used for this study were group-housed in the animal facility of the Institute of Molecular Psychiatry under standard conditions (12 h light/dark cycle, lights on at 7 a.m., tap water and food ad libitum, room temperature 22 °C, humidity 48%).

### 2.3. ^15^N Metabolic Labeling of CD1 Mice

The ^15^N metabolic labeling of CD1 mice was performed as previously described [9], according to the protocol established by Frank et al. [10], using a ^15^N-labeled, bacterial, protein-based diet (Silantes GmbH, Munich, Germany) which results in > 91% ^15^N incorporation both in mouse brain tissue and plasma [11].

### 2.4. Sample Collection

At eight weeks of age, hippocampi from G72Tg, WT and ^15^N-labeled CD1 animals were dissected after perfusion with 0.9% saline. Hippocampi were snap frozen in liquid nitrogen and stored at −80 °C.

### 2.5. Quantitative Proteomics Sample Preparation and Measurement

The hippocampal cytoplasmic fraction from G72Tg, WT and ^15^N-labeled CD1 mice was collected as previously described [9]. ^15^N-labeled CD1 mouse hippocampal tissue was used as internal standard so as to compare the unlabeled G72Tg and WT mice. The experimental design for the quantitative proteomics analysis is as previously described (Figure 1 in [9]). The Bradford assay (BioRad Laboratories, Hercules, CA, USA) was used to quantitate total protein amount. For each G72Tg/WT pair, the cytoplasmic fractions of G72Tg and WT hippocampi were mixed 1:1 (*w*/*w*) with the cytoplasmic fraction of the same ^15^N-labeled CD1 mouse reference based on protein amount. Proteomics sample preparation for sodium dodecyl sulfate-polyacrylamide gel electrophoresis (SDS-PAGE), in gel digestion and peptide extraction was performed as previously described [9,10]. Peptide extracts were lyophilized, re-dissolved in 0.1% formic acid, filtered and analyzed using liquid chromatography-electrospray ionization-tandem mass spectrometry (LC-ESI-MS/MS) by a nanoflow HPLC-2D system (Eksigent, Dublin, CA, USA) which was coupled online to an LTQ Orbitrap XL™ Hybrid FT Mass Spectrometer (Thermo Fisher Scientific, Bremen, Germany), as previously described [9]. Detailed MS parameters were as previously described [12].

### 2.6. Quantitative Proteomics Data Analysis

Quantitative proteomics data analysis was performed based on established workflows [9,13], including the Trans-Proteomic Pipeline [14] in order to identify protein groups [15]. Protein quantification results were manually evaluated to exclude inaccurate quantifications due to incorrect isotopologue pattern assignment or protein contaminants. Protein groups with adjusted *p* < 0.05 were considered differentially expressed, however, only protein groups with a fold change > 1.3 were considered biologically relevant and were further discussed. MS raw data and the quantification analysis result files are available upon request.

### 2.7. Protein Network Visualization

To visualize altered protein networks between G72Tg and WT hippocampi, we used the list of differentially expressed proteins as input for STRING (https://string-db.org, v.11.5). *Mus musculus* was selected as the organism and the default settings, including both functional and physical protein associations, were kept in order to identify protein networks.

### 2.8. Targeted Metabolomics

Sample preparation for metabolomics of G72Tg and WT hippocampi (*n* = 6 per group) was performed as previously described [16,17]. Samples were measured with a 5500 QTRAP^®^ triple quadrupole mass spectrometer (AB/SCIEX, Framingham, MA, USA) which was coupled to a Prominence UFCL HPLC system (Shimadzu, Columbia, MD, USA) at the Mass Spectrometry Core of Beth Israel Deaconess Medical Center, Harvard Medical School (Boston, MA, USA), using a selected reaction monitoring (SRM)-based platform [18].

### 2.9. Targeted Metabolomics Data Analysis

Metaboanalyst 5.0 (https://www.metaboanalyst.ca) [19] was used to analyze targeted metabolomics data. For metabolites which were measured both in positive and negative ion mode, the measurement with higher intensities/fewer missing values was kept for further analysis. Hippocampal metabolite raw peak intensity data that were used for Metaboanalyst are provided in Table S1. Metabolites with > 10% missing values were not considered for further analysis and the remaining missing values were replaced by the 1/5 of the minimum positive value of each variable. No data filtering was applied. Data were then median-normalized, log-transformed and Pareto-scaled. Data were assessed using a univariate method (significance analysis of microarrays (SAM)). For SAM analysis, features with false discovery rate (FDR) < 0.05, *p* < 0.05 and *q* < 0.05 were considered significant.

### 2.10. Western Blot

G72Tg and WT mouse hippocampal cytoplasmic Mog, Cldn11, Plp protein expression (*n* = 10 per group) was assessed using anti-Mog (ab28766, Abcam, Cambridge, UK, 1:15,000 dilution, 10 μg loaded per sample), anti-Cldn11 (Osp) (ab53041, Abcam, 1:5000 dilution, 2 μg loaded per sample) and anti-Plp (ab28486, Abcam, 1:5000 dilution, 2 μg loaded per sample) primary antibodies, respectively. Western blot was performed as previously described [12]. Transfer membranes were stained with Coomassie Brilliant Blue R-250 (BioRad Laboratories, Hercules, CA, USA) and signal intensities were compared to ensure equal protein loading. For signal intensity quantification QuantityOne (version 4.4.0, BioRad Laboratories, Hercules, CA, USA) was used. G72Tg and WT group differences in Western blots were assessed by the non-parametric, Mann–Whitney test using GraphPad Prism 8.0 (GraphPad Software, San Diego, CA, USA). Results were considered statistically significant for *p* < 0.05.

## 3. Results

### 3.1. Decreased Expression of Myelin-Related Proteins and Increased Expression of Presynaptic Proteins in G72Tg Hippocampi

**Figure 2:**
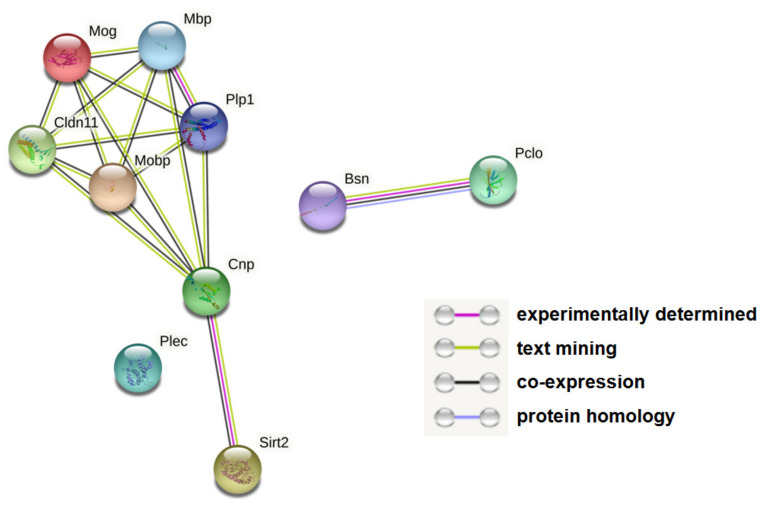
Altered protein networks in G72Tg compared to WT hippocampi. Each node represents a protein. Lines connecting the nodes indicate functional and physical associations between the respective proteins. Line color denotes the type of evidence for the reported association, as indexed in the figure. Full protein names are listed in Table 1. The figure was generated by STRING (https://string-db.org, v.11.5).

In this study, we explored molecular changes in the hippocampi of G72Tg compared to WT mice using quantitative proteomics and metabolomics. Our quantitative proteomics analysis revealed 14 proteins (protein groups) that were differentially expressed between G72Tg and WT mouse hippocampi, of which 10 were considered statistically and biologically significant (Table 1). Of these, the expression of seven proteins was lower and of three proteins was higher in G72Tg compared to WT mice. Except Sirt2, proteins with decreased expression were predominantly related to myelin (Plp1, Mbp, Cnp, Mog, Mobp, Cldn11). Proteins with increased expression in G72Tg hippocampi were presynaptic, namely the scaffold proteins Pclo and Bsn, as well as cytoskeletal (protein Plec1). In silico analyses revealed physical and functional associations among the proteins with decreased expression in G72Tg hippocampi as well as between the two presynaptic scaffold proteins (Figure 2).

### 3.2. Decreased Nicotinate Levels in G72Tg Hippocampi

**Figure 3:**
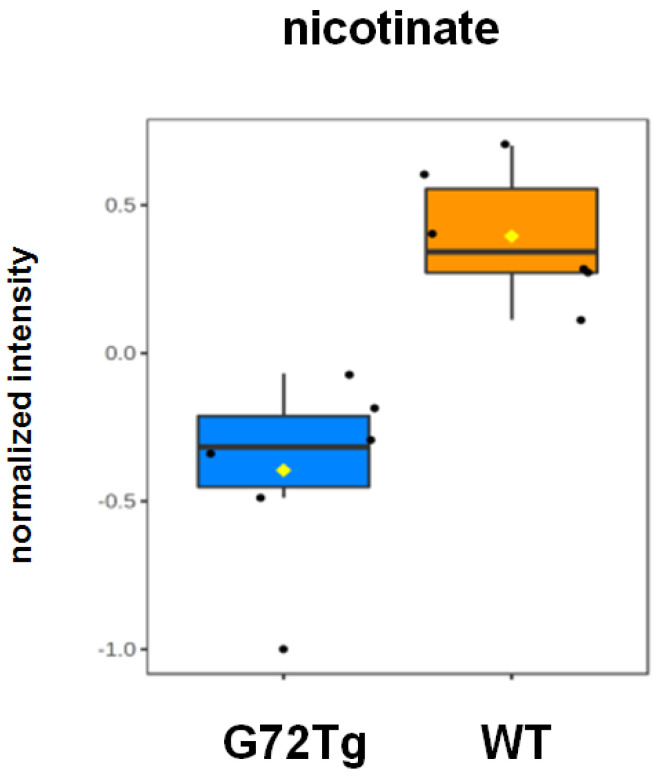
Decreased nicotinate levels in G72Tg compared to WT hippocampi. FDR: 0.015, *p* = 0.000184 *q* = 0.0206. Data are presented as box and whisker plots (*n* = 6 per group).

Using an SRM-based, targeted metabolomics platform, 289 metabolites were measured in G72Tg and WT hippocampi, of which 273 were kept for further analysis, after including only once metabolites that were measured in both negative and positive ion modes (Table S1). SAM analysis identified nicotinate (conjugate base of nicotinic acid, termed niacin or vitamin B3) as a significant feature. Nicotinate levels were lower in G72Tg compared to WT hippocampi (Figure 3).

### 3.3. Convergent Protein Expression Changes in Hippocampus and Cerebellum in G72Tg Mice

**Figure 4:**
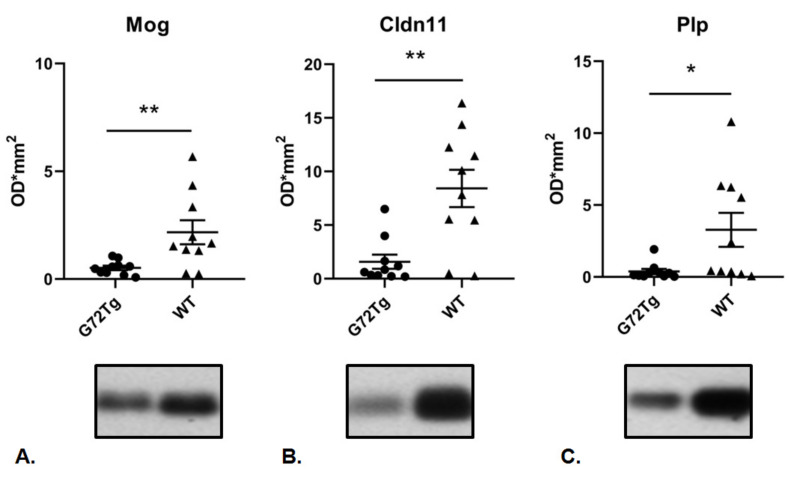
Decreased expression of myelin proteins in G72Tg compared to WT hippocampi. Decreased expression of (**A**) Mog (** *p* = 0.0089) (**B**) Cldn11 (** *p* = 0.0089) and (**C**) Plp (* *p* = 0.0147) in the cytoplasm of G72Tg (*n* = 10) compared to WT (*n* = 10) hippocampi. Data are presented as mean ± standard error of the mean (SEM).

To investigate whether there are common G72Tg-induced molecular patterns in different mouse brain regions, we then compared the differentially expressed proteins identified in the hippocampus cytoplasm of G72Tg mice (Table 1) with the previously identified differentially expressed proteins in the cerebellum cytoplasm of G72Tg mice ([9], Table 1). Three proteins, Plp, Cldn11, and Mog, were found at lower levels both in the cerebellum and hippocampus in G72Tg compared to WT mice. Their decreased expression was verified in a larger number of male G72Tg and WT mouse hippocampi (*n* = 10 per group) by Western blot analysis (Figure 4). Full Western Blot data are provided in Supplementary Figure S1.

## 4. Discussion

In this study, we used MS-based quantitative proteomics and metabolomics to illuminate G72-induced molecular alterations in the hippocampi of G72Tg male mice. We selected the hippocampus as a brain region of interest because in previous studies, next to cerebellum and cortex, it was shown to have the highest G72 mRNA expression levels [6]. Furthermore, the hippocampus has been implicated in the pathophysiology of schizophrenia [20,21]. Overall, our multi-omics analysis revealed decreased expression of myelin-related proteins and increased expression of presynaptic scaffolding proteins, along with lower levels of nicotinate in G72Tg compared to WT hippocampi.

Hypomyelination has been reported in schizophrenia, both in rodent models [9,22] and human cohorts [23]. Convergent evidence indicates altered expression of myelin-related proteins in schizophrenia patients, in different brain regions, such as the thalamus [24], the corpus callosum [25] and the dorsolateral prefrontal cortex [26]. In the hippocampus, decreased Mog protein expression in CA3 was observed in patients with long-term schizophrenia [27], along with decreased oligodentrocyte numbers in the left CA4 in patients with schizophrenia [28] compared to controls. In our efforts to characterize lipid-based, myelin-related changes in the hippocampus, we undertook a lipidomics study to compare G72Tg and WT mice and found increased sulfatide levels in G72Tg hippocampi [29]. Interestingly, in frontal cortex samples of patients suffering from schizophrenia analyzed with the same lipidomics platform also elevated sulfatide levels were identified [29]. In our proteomics analysis here, we observed decreased Sirt2 and Cldn11 expression in G72Tg hippocampi. Although there is limited information on a potential role of these two proteins in schizophrenia, altered phosphorylated Sirt2 protein expression was reported in response to the antipsychotic clozapine in rat nucleus accumbens [30], whereas myelin mutant mice lacking Cldn11 expression showed changes in behavior and neurotransmitter level imbalance [31].

We also observed increased protein expression of the scaffold presynaptic proteins Pclo and Bsn in G72Tg hippocampi. Synaptic alterations have been extensively described in schizophrenia [32], including changes in presynaptic neurotransmitter release [33] and post-synaptic characteristics [34]. However, there is limited information on a potential implication of Bsn and Pclo in schizophrenia manifestation. Recently, mutations in both Pclo and Bsn genes have been identified in subjects with schizophrenia [35], whereas increased Pclo gene expression was reported in the amygdala of schizophrenia patients [36]. Furthermore, mice with suppressed Pclo expression in the medial prefrontal cortex exhibited schizophrenia-like behaviors [37]. Intriguingly, in an interactome study of D-amino acid oxidase (DAO) in the rat cerebellum, both Bsn and Pclo were identified as DAO interacting proteins, and Bsn was shown to co-localize with DAO and inhibit its enzymatic activity [38], suggesting that Bsn may have a regulatory role in downstream, G72 (DAOA)-related pathways.

Metabolomics is a powerful approach to detect altered biosignatures in psychopathologies [39], which we have implemented to disentangle molecular profiles of neuropsychiatric phenotypes [40,41,42,43] as well as responses to pertinent therapeutic interventions [44,45,46,47,48,49,50]. Here, we used a targeted metabolomics platform to compare G72Tg and WT mice and identified lower nicotinate levels in G72Tg hippocampi. Nicotinate (in the form of niacin/vitamin B3) is the precursor of NAD^+^-NADH and NADP^+^-NADPH electron carriers. Schizophrenia risk has been associated with an enzyme involved in nicotinate metabolism in a genome-wide association study of an Indian population [51], whereas a skin flush response to niacin has been discussed as a potential marker for a schizophrenia endophenotype [52].

We have previously found that LG72 localizes in mitochondria both in human and murine cell lines and that LG72 interacts in both cases with the mitochondrial protein methionine sulfoxide reductase B2 (MSRB2) [53]. Importantly, the LG72 protein has been shown to disrupt mitochondrial functions [7]. Mitochondria are highly conserved organelles across mammals and mitochondrial dysfunction has emerged as a core molecular pathology in psychiatric disorders, including schizophrenia and bipolar disorder [54,55]. Due to the fact that in vivo studies of this kind in humans and non-human primates are difficult, if not impossible, our G72Τg mouse model provides a valid system for the study of G72 function in vivo.

## 5. Conclusions

Taken together, our multi-omics approach revealed altered molecular networks in the hippocampi of G72Tg compared to WT mice which shed light on the underlying G72-dependent molecular mechanisms. The identified molecular changes may be used in drug development efforts for the treatment of schizophrenia.

## Figures and Tables

**Table 1:** Differentially expressed proteins in the hippocampi of G72Tg and WT mice (fold change > 1.3, adjusted *p* < 0.05).

Protein	Protein Name	G72Tg/WT Fold Change	Adjusted *p*-Value	Protein Groups
Decreased expression in G72Tg hippocampi				
Mobp	Myelin-associated oligodendrocyte basic protein	0.50	4.88409 × 10^−5^	MOBP_MOUSE
Plp1	Myelin proteolipid protein	0.54	8.06522 × 10^−81^	Q3UYM8_MOUSE, MYPR_MOUSE
Cldn11 (Osp)	Claudin-11, oligodentrocyte-specific protein	0.54	1.72045 × 10^−9^	CLD11_MOUSE
Mbp	Myelin basic protein	0.57	2.16222 × 10^−24^	Q542T4_MOUSE
Mog	Myelin oligodentrocyte glycoprotein	0.57	1.26402 × 10^−9^	MOG_MOUSE, Q80YU5_MOUSE, Q3UY21_MOUSE
Cnp	2′,3′-cyclic-nucleotide 3′-phosphodiesterase	0.66	8.69626 × 10^−23^	CN37_MOUSE, Q3TYV5_MOUSE
Sirt2	NAD-dependent protein deacetylase sirtuin-2	0.71	0.001562104	SIRT2_MOUSE, Q3UJK6_MOUSE
Increased expression in G72Tg hippocampi				
Bsn	Protein bassoon	1.41	1.34696 × 10^−8^	BSN_MOUSE
Plec1	Plectin	1.41	0.015085062	Q6S387_MOUSE, Q6S390_MOUSE, Q6S388_MOUSE, Q6S385_MOUSE, Q6S392_MOUSE, PLEC1_MOUSE, Q6S393_MOUSE
Pclo	Protein piccolo	1.74	0.00012123	PCLO_MOUSE

## Data Availability

The raw data presented here are available on request from the corresponding authors.

## Supplementary Materials

The following supporting information can be downloaded at: https://www.mdpi.com/article/10.3390/jpm12020244/s1, Table S1: Raw hippocampal metabolite data of G72Tg and WT mice considered for metabolomics analysis. Figure S1: Full Western blot data of Mog, Cldn11 and Plp using G72Tg and WT mice hippocampal extracts.
