# Supplementary material for: Multi-Omics Analysis Reveals Myelin, Presynaptic and Nicotinate Alterations in the Hippocampus of G72/G30 Transgenic Mice
Source: J Pers Med. 2022 Feb 9;12(2):244. doi: 10.3390/jpm12020244 (PMC8878587; doi:10.3390/jpm12020244)
Supplement: Supplementary file 1 [file jpm-12-00244-s001.zip › jpm-1546187-supplementary.pdf]

Journal of *Personalized Medicine*

Supplementary Materials

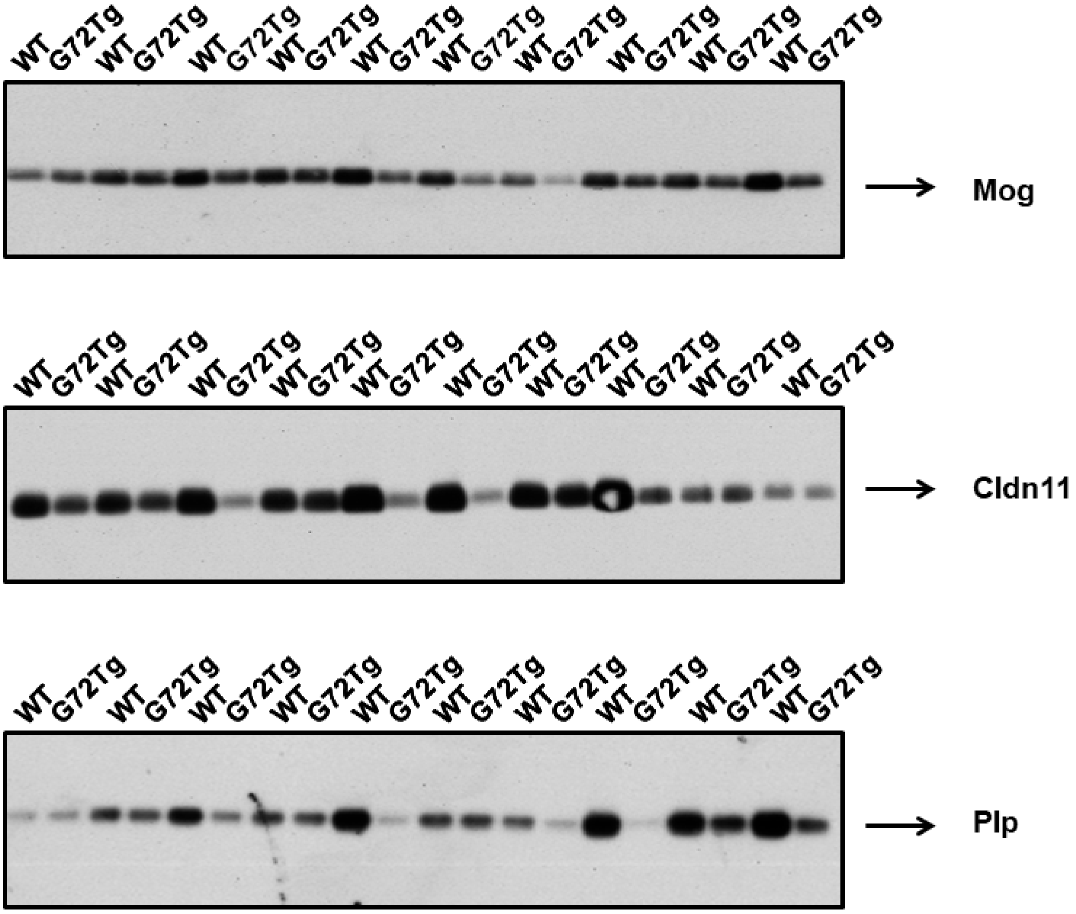

Figure S1. Full Western blot data of Mog, Cldn11 and Plp using G72Tg and WT mice hippocampal extracts.

Table S1. Raw hippocampal metabolite data of G72Tg and WT mice considered for metabolomics analysis.

Sample	HIP847	HIP850	HIP915	HIP916	HIP918	HIP919	HIP848	HIP849	HIP902	HIP904	HIP917	HIP942
**Metabolite**	**G72**	**G72**	**G72**	**G72**	**G72**	**G72**	**WT**	**WT**	**WT**	**WT**	**WT**	**WT**
1,3-diphopshateglycerate	489588.4856	NA	NA	NA	NA	NA	NA	NA	NA	NA	NA	NA
1-methyladenosine	8484889.586	5389677.27	4535024.175	2546503.19	5677379.895	2822281.851	3432989.219	8683247.275	6493984.525	4163150.196	1949136.542	1195639.977
1-methyl-histidine	830285.235	950195.4381	1089152.495	774994.9423	1429584.201	1133781.313	692349.6105	972785.6242	1268456.948	1142385.97	693568.5678	521549.5466
2,3-dihydroxybenzoic acid	804496.3252	458777.1382	339471.0734	145717.7654	285251.1425	211832.2067	38149.25458	433613.6522	213446.3499	291486.04	120366.866	147142.9259
2,3-diphosphoglyceric acid	201781.6759	19478.86699	NA	NA	11341.21684	NA	22271.8656	13419.55831	29543.8351	NA	NA	NA
2-aminooctanoic acid	13302223	14993201.26	5722098.952	6310497.343	7035164.475	3498877.022	14855930.36	26467538.7	5048460.682	16619631.52	6238360.564	2098144.697
2-dehydro-D-gluconate	31374.45467	17610.75823	20947.81213	10508.89817	27214.08905	NA	21207.97943	15928.93006	NA	8748.563665	16671.80386	16023.37192
2-deoxyglucose-6-phosphate	23168492.59	9795125.53	10545007.32	4919020.421	11382299.38	6518667.788	7571772.587	8221603.3	13017969.93	8127730.277	4400880.862	3572982.084
2-hydroxy-2-methylbutanedioic acid	1003538.488	617727.6208	402297.3677	422708.5708	574652.5434	417761.2139	533195.9406	562545.8418	498005.6888	898495.2818	327396.1828	551323.8418
2-hydroxygluterate	2913085.142	1864315.088	1532287.644	1137673.851	2218707.591	873676.3595	1158794.35	1499345.085	1552979.937	1828820.83	682671.4672	667630.4098
2-isopropylmalic acid	106889886.8	95733156.81	85076879.87	75910420.89	99993811.71	83836579.71	122989090.3	87146505.25	79048455.91	71665982.91	76813878.22	105543014.7
2-ketohaxanoic acid	97842.72877	15848.92914	NA	40313.20208	44772.5034	9416.313466	178500.7601	40466.80152	31072.46783	41878.53452	45792.44341	34862.22328
2-keto-isovalerate	13418005.81	8772607.981	8371186.408	7058299.073	9667288.472	7321090.796	9794191.897	8366416.726	6817318.74	5538889.321	8688242.728	9542162.821
2-oxo-4-methylthiobutanoate	90787.31185	109606.4293	20393.09617	99726.18362	70342.73438	90436.16438	92872.52298	114857.9531	49246.73763	27864.0411	90767.65195	76103.88439
2-oxobutanoate	322623.9113	190122.215	224127.8534	187763.447	310486.3716	222078.8007	239387.0404	109574.6768	159391.1594	218497.1078	197131.0751	257830.0119
3-hydroxy-3-methylglutaryl-CoA	NA	NA	NA	NA	NA	NA	NA	NA	NA	NA	NA	NA
3-hydroxybutyryl-CoA	NA	NA	NA	NA	NA	NA	NA	NA	NA	NA	NA	NA
3-methylphenylacetic acid	16753.44205	NA	NA	NA	13133.68264	7842.665117	19220.15461	NA	NA	NA	NA	24564.05523
3-phosphoglycerate	2578310.777	827900.0744	243129.6394	38001.09198	301054.6756	59843.74048	841267.9603	250540.0688	1074941.409	104727.0146	351128.3854	119008.9666
3-phospho-serine	NA	NA	6337.540157	10993.37562	10854.6804	15112.39327	21803.22983	8231.631461	9140.477311	12353.09479	7269.788678	NA
3-S-methylthiopropionate	13801.10868	NA	12868.26608	NA	13462.75499	11912.53783	NA	NA	NA	NA	NA	7251.997611
4-aminobutyrate	51578887.84	46349630.17	45162525.83	31819747.23	53367020.51	35113272.92	31615548.38	40974712.85	51991414.31	48310176.99	24136910.43	19290782.95
4-pyridoxic acid	291365.592	119484.121	327786.7659	102060.2687	213312.8723	52598.66478	25572.98994	115614.7317	164784.7462	195835.8084	52291.43705	44328.10625
5-methoxytryptophan	NA	NA	NA	NA	NA	8236.569412	NA	NA	NA	NA	NA	NA
5-methyl-THF	26901.65393	44073.8653	20268.43926	46680.49016	39841.67685	29496.23313	18803.47193	38397.58401	19455.3995	29624.71354	12890.66184	12084.59954
5-phosphoribosyl-1-pyrophosphate	93869.49401	71515.50643	82986.39235	52566.15918	57676.69794	42564.17717	46416.28343	62642.56808	62619.95587	47704.80744	29840.77811	21704.62517
6-phospho-D-gluconate	181453.0522	186085.3739	72014.79882	63024.65709	106922.9912	41847.78399	117309.0414	89795.37571	232195.2786	129220.6803	48657.97308	36115.74512
7,8-dihydrofolate	NA	NA	NA	NA	NA	NA	NA	NA	NA	NA	NA	NA
7-methylguanosine	185451.249	113824.1626	89565.53148	93187.61206	127351.7327	69810.82395	67805.92544	97234.31202	122663.3319	87284.39042	43862.01135	36757.38367
acadesine	21263.24807	26579.58058	18336.98111	6948.152298	24843.30484	NA	8817.443971	NA	12809.38397	NA	NA	12358.02738
acetoacetate	337056.7203	259876.6231	169419.8607	159056.2677	256230.9531	217901.6079	252111.4068	165806.994	132055.728	139479.0252	147083.8603	334853.4101
acetoacetyl-CoA	47626.84134	31051.16202	12941.87342	10347.75782	27435.84901	20251.63405	22370.26656	15530.41007	21224.30421	17604.73656	16566.78256	5695.346103
acetylcarnitine DL	125217149.2	122915926.7	119393964	128689594.2	131448893.4	133502722.5	127592701.1	141940432.1	127536949.1	113349459.9	115822313.2	124282317.6
acetyl-CoA	937578.0449	182425.0241	384776.6475	289904.4975	218259.1068	368996.5054	277174.9561	205559.7241	348523.8431	166486.6628	216098.1694	340410.9638
acetyllysine	33775702.63	17023499.36	11200726.31	8412664.469	22283285.22	6842491.362	10562073.27	13209605.61	27392394.79	21007198.99	8312496.158	2848166.289
acetylphosphate	658567.7992	481400.9473	383761.031	238124.292	510097.2981	387362.7481	347615.402	408527.2247	468191.258	378547.5975	246500.9223	269543.9903
aconitate	306372.947	334223.2641	385855.5234	396742.7376	388874.5296	299373.2078	308952.7514	371598.3826	252618.2002	192989.8442	313829.266	524670.8854
adenine	14229644.89	9260072.333	6764570.861	5042824.044	8785400.087	6315491.19	5002646.941	8361086.746	10982006.09	7131104.07	4239335.037	2515758.097
adenosine	1928320.558	1060163.898	1241287.305	885074.6665	1301855.044	897096.752	852738.3335	964054.6819	3524168.318	1069692.735	626374.7415	397200.9913
adenosine 5-phosphosulfate	145859.9676	117002.6192	100926.2652	71959.83617	105908.9504	79693.40374	88628.49972	99869.82609	75614.58194	72481.36454	74018.42005	47498.6125
ADP-D-glucose	47722.69207	15014.92664	16047.83695	15461.8972	30603.33303	18642.46686	16565.92126	19154.38107	15448.44108	21229.99437	15531.60329	8805.259077

ADP	23709108.57	16947081.55	12914863.13	10569494.8	15938047.5	12172139.66	12827600.27	13619008.89	10710619.4	6797512.674	9924110.75	6813952.254
a-ketoglutarate	517894.9448	209772.499	175794.992	94737.61602	469519.7828	91146.23703	426350.9054	303428.9501	222733.7461	164318.0959	177841.5878	452837.1159
alanine	53977172.75	42895261.72	44022244.72	40969261.81	51028394.42	38649350.58	34164998.39	43188597.57	56349161.44	48637104.85	29086749.06	25269795.83
allantoate	162817.5804	102379.2222	78008.94825	70292.77551	99076.94779	51531.58396	115022.9062	90684.9184	67481.08537	41101.35021	94323.59916	71623.51261
allantoin	537219.1737	339624.9197	206211.9469	120167.6565	244694.0494	124945.4823	156836.7884	180817.9416	190598.2811	164541.2076	134321.0791	167924.3506
aminoadipic acid	375872.2758	158078.2827	103175.2171	97318.70331	228044.2588	107746.786	175002.95	202763.9276	114156.1268	99965.79706	128795.0816	168287.5514
aminoimidazole carboxamide ribonucleotide	10814.8103	23540.32519	32467.39551	NA	25844.34247	29510.39499	13173.4042	18473.41933	19183.05218	9699.708433	24799.98708	18156.31744
AMP	133444573.2	86442808.37	113371726.7	122317004.8	78205984.19	113313464.9	105518452.2	123171110.9	125847273.5	117207585.1	114456134.5	101424730.3
anthranilate	984629.3032	692267.2723	440673.5792	371490.8158	756213.5686	235635.2734	290848.5525	531404.2943	793294.7663	789685.876	322403.4107	156062.4727
arginine	22478861.46	13947768.99	13550644.43	8016398.715	17036213.93	8843147.831	6423772.912	11307422.58	24224029.32	16983324	4405452.125	5580571.999
ascorbic acid	87779553.68	58755552.1	48652384.34	41871041.98	59664285.27	45083393.88	76500682.84	51834919.29	42259106.17	36309040.62	42976239.24	58266577.81
asparagine	4039264.266	3252784.64	4037819.943	2567768.47	3720682.008	3227180.466	1903130.1	3064335.107	3926337.009	3467516.872	2438250.942	1658533.402
aspartate	18820847.1	28086643.73	29334957.03	23224375.2	27025243.98	21402059.9	23037545.6	26584699.26	18695703.84	27403793.7	25873629.51	15956117.46
ATP	9039870.908	7168939.803	5227335.825	3677557.141	6741674.426	5676749.442	4155756.475	4897089.287	7602306.58	3279255.06	4416372.262	2175990.189
atrolactic acid	281353.717	266094.8698	175723.0345	195385.5215	226062.8794	71798.81922	123400.8604	127055.1361	161928.357	137453.447	121017.3463	68301.00465
betaine	49848328.56	54479138.59	40972617.28	50391124.23	38708529.47	52658182.51	75492795.82	66910666.89	47806801.29	58362835.24	58338953.07	67729551.85
betaine aldehyde	230246.0146	194123.6251	217994.2758	205642.4851	250540.1021	183630.1185	269371.6279	196149.7701	225843.8462	200029.1465	200199.0432	236667.8735
biotin	93826.21424	NA	NA	NA	79258.34497	65782.39457	NA	631109.5119	NA	NA	NA	49944.68005
butyryl-CoA	NA	NA	NA	NA	NA	NA	NA	NA	NA	NA	NA	NA
carbamoyl phosphate	128277998.6	103949056.5	103457012	71684204.94	110495708	79298164.03	77641924.51	99465243.76	93488768.77	79466462.23	82836121.45	60011532.05
carnitine	40766639.99	41077678.82	54155398.24	37386162.06	48754719.43	58360403.83	55784354.23	45629044.85	46287334.75	41422080.22	50276143.4	50247418.65
CDP-choline	314197.1588	177970.1779	169081.5022	96290.62414	207880.7459	119867.3879	208892.6866	195211.6703	160163.4331	132041.8569	262308.6924	225683.3703
CDP-ethanolamine	533068.973	316813.8661	297856.7782	276998.2453	414815.873	259972.565	276450.7332	285138.2349	326707.7901	215337.633	268206.7776	241191.2063
CDP	144690.5633	188125.7243	98620.32699	77038.15667	83028.50954	86245.12477	103984.0293	128306.2703	93875.45274	69278.7812	48187.62465	61334.14337
cellobiose	12504.5048	11859.97173	33266.47138	NA	11387.25574	8821.889822	20285.18609	21829.50822	17194.88439	10506.9316	8322.897298	15984.2193
cholesteryl sulfate	287276.1078	287883.1307	128847.0199	488715.0505	271371.2869	169761.3103	110314.5372	179906.8044	1566442.662	783950.0564	54875.6471	77136.58467
cholic acid	NA	NA	NA	NA	NA	NA	NA	NA	NA	NA	NA	NA
choline	1170180.507	402733.4546	299789.8636	NA	821711.7687	NA	NA	1008497.19	687280.0603	NA	NA	480954.2622
citraconic acid	63610.26605	20005.31396	52100.08408	189948.3803	60694.83914	52967.84366	112546.3108	22681.12158	240498.0119	100508.9398	44346.12779	94620.02131
citrate	22858086.34	18626808.39	16066112.89	12454920.84	15417690.35	13330032.94	20964073.89	17373376.49	13644623	11028128.45	10095109.43	15075186.57
citrate-isocitrate	52182666.56	45885977.3	35852388.92	26546783.4	34834043.6	29255874.26	50130656.03	32985480.64	30706013.56	22489988.28	24699222.32	35236820.88
citrulline	963740.4082	1095158.536	899627.3324	899585.5683	1122403.268	1044953.344	656767.0858	920032.7669	1211603.525	944588.4042	860939.1028	875609.837
CMP	3019381.884	2381218.029	2766830.245	3159724.823	3238952.413	2964223.955	2226107.898	2968789.274	3208291.468	2663986.482	2324403.535	2393079.973
coenzyme A	4226568.059	955688.0683	1218575.575	364311.888	1803366.023	1885257.302	2144574.242	1547687.029	1745790.016	465124.9642	1383685.922	1892131.516
creatine	315613083.1	285955876	298846391.8	274911076.9	311878504.1	249184745.2	239866590.5	329256207.6	326310881.2	285873679.6	210911766	187651611.8
creatinine	19833993.84	12934975.4	13115547.14	10612978.02	17114411.75	9821228.978	7831809.989	17701737.93	18549889.07	15590911.28	6113209.513	5116151.829
CTP	286414.6251	176186.3491	91484.52751	83309.40099	171352.8885	159390.7006	101416.6	155309.301	198250.2974	110241.7782	46333.61138	68902.37568
cyclic bis(3->5) dimeric GMP	NA	NA	NA	NA	NA	NA	NA	NA	NA	NA	NA	NA
cyclic-AMP	3236196.833	1226257.92	943083.3304	443924.8089	1222599.309	388471.0716	669169.1442	1044265.574	1543233.094	550363.3018	564036.3411	339428.8097
cystathionine	644645.8886	749381.5802	532679.1495	542345.6839	617600.7483	638855.8803	712785.1905	912083.6897	770608.4363	480141.3604	232653.8817	584352.931
cysteine	156020.0452	23491.94456	25355.95699	NA	30924.11593	39450.5796	68317.44018	48288.21822	NA	21559.03536	38456.29102	35571.06851
cystine	51213.18905	37579.33366	11962.71662	21797.10841	29230.7058	31361.15483	26899.95087	11799.97405	44993.46244	27885.85743	29760.61382	18385.59541
cytidine	3954012.122	392425.8156	304534.45	1543884.124	352298.916	441140.7124	2409976.914	391346.6868	3687668.126	895629.6606	442721.2615	1111462.096
cytosine	NA	110913.6914	107755.9746	41440.49127	134810.7713	65901.58807	95761.8129	158994.7547	112703.0959	109430.7298	69403.68196	NA
dAMP	437726.5012	341930.9951	355090.4374	220136.5595	390076.9239	292347.2986	417790.6406	444591.1858	371871.0247	247500.0608	519133.6266	158926.1611

dATP	NA	NA	NA	NA	NA	NA	NA	NA	NA	NA	NA	NA
dCDP	10353.6726	12423.57914	NA	8284.321195	17605.26895	7246.547278	8283.057358	10333.62773	NA	NA	NA	NA
dCMP	32612.06639	33626.49107	37767.95115	29507.07187	49698.54446	32397.32755	47500.49103	42485.66983	39863.31653	35666.60426	23291.03053	26569.88429
dCTP	NA	NA	NA	NA	NA	NA	NA	NA	5695.407983	NA	NA	NA
deoxyadenosine	NA	NA	NA	NA	NA	NA	NA	NA	NA	NA	NA	86717.15367
deoxycholic acid	22151.35548	15013.28347	6728.276459	NA	NA	2541800.406	20645.20227	7760.082038	NA	6219.030467	9837.123397	NA
deoxyguanosine	53153.92229	48525.39258	47326.47387	32508.29745	25576.44437	27405.01253	38872.72832	28768.36495	45452.09309	36175.62559	19128.45235	11431.0761
deoxyinosine	NA	NA	NA	NA	14178.78926	NA	NA	9400.95122	7856.696698	NA	NA	NA
deoxyribose-phosphate	293046.3731	114135.1799	118380.8128	170819.214	74877.71651	120556.1975	163560.1093	95404.92661	94648.16148	139192.6224	136624.495	104354.7807
deoxyuridine	112853.9906	29000.19206	15530.9029	NA	37792.12561	10397.92541	21219.44728	23526.77256	22779.44631	14496.21509	13484.68099	26281.54693
dephospho-CoA	1460859.012	601168.9187	432268.4201	781673.0727	892440.2277	1108480.005	587064.4208	814599.582	944327.3993	788546.988	366770.5115	1199208.785
D-erythrose-4-phosphate	1924381.45	1043159.627	790788.8678	474910.2209	766020.9572	524186.6272	539952.9492	663004.3061	669057.5847	500282.9046	394058.6884	286134.9057
dGDP	22220886.59	16429145.36	13889231.18	10255925.84	16147532.04	12626259.22	13175058.75	13998493.11	11055061.12	7205702.95	10425410.43	7347423.47
D-glucarate	NA	62231.11356	58001.29894	77951.91288	50279.43061	54539.44294	36721.93813	43897.41635	27705.14684	49236.49039	NA	46697.38339
D-gluconate	215601.2483	119067.6171	75572.6516	68287.63484	99580.22438	53122.1614	65927.30625	88338.32553	74724.77828	55149.07424	35170.6156	58074.26832
D-glucono-1,5-lactone-6-phosphate	86472.80297	74576.58131	35844.72431	15448.63035	56565.90462	34181.81291	29816.65698	22710.05574	8045.492754	37752.61042	23133.93471	9285.946431
D-glucosamine-1-phosphate	25872.97994	18637.79638	38228.69402	23287.435	35609.94224	25118.38487	25837.35261	24949.63872	20644.0754	29954.09912	27419.92354	17685.43028
D-glucosamine-6-phosphate	19890.84725	31733.1525	23955.24711	17535.1688	13981.39776	15297.25534	12892.06074	24849.39044	28427.30755	22687.4191	16572.68158	NA
D-glyceraldehdye-3-phosphate	1210966.991	4162299.929	1361155.815	1352590.079	2030950.171	3135516.125	419488.3875	1827416.09	1386409.115	1722695.992	1200336.356	1296004.682
dGMP	64592.68912	35379.44821	44345.04203	37009.71049	34972.09232	17604.84142	14744.88096	46563.61464	25968.87285	27452.74692	34103.65391	18332.24873
dGTP	8997120.563	6920202.993	5001710.037	3600112.964	6310231.548	5676018.281	3756436.122	4519587.723	7174687.364	3401997.755	4380778.152	2140344.471
dihydroorotate	498874.1863	291122.9574	250522.221	248368.6553	265620.2959	247137.0842	309405.0548	273484.6654	134220.3251	212374.3302	307785.5298	503700.5511
dihydroxy-acetone-phosphate	2453019.591	5824435.732	2007540.608	2092551.107	2846912.382	4000621.263	785078.7547	2671993.125	2400569.816	2810416.699	1692407.221	1814457.436
diiodothyronine	NA	NA	NA	NA	NA	NA	NA	NA	NA	NA	NA	NA
dimethylglycine	854285.5148	786650.3861	530905.5989	522647.6382	868830.4929	566609.1445	449425.9016	675013.6358	645164.3645	848624.3521	324878.3628	339628.9464
DL-pipecolic acid	103104161.4	72144597.56	3359516.96	69442409.84	84287633.7	57102761.6	55436696.44	72383215.24	98776310.26	77858825.68	52256832.19	43570241.74
D-sedoheptulose-1-7-phosphate	1701407.396	1432663.44	628467.9484	422837.6968	829956.5619	656948.7506	750398.0649	645117.2475	834928.2654	670855.4464	587641.036	709966.7323
dTDP	38826.18499	16565.22868	10916.49447	14498.37868	25369.34327	10873.05557	NA	6732.8336	8280.774739	10353.97416	NA	NA
dTMP	94067.82115	79120.04164	42449.85952	77135.98741	58374.08856	49699.92328	26893.63664	28268.63412	86941.00857	33916.64259	6087.335872	13939.6678
dTTP	NA	NA	NA	6212.171775	NA	7248.443037	NA	NA	8801.26632	NA	NA	NA
dUMP	226236.7382	89080.55094	69883.57366	68908.0868	102275.4376	54364.51555	31055.41572	64715.07644	113396.7482	62126.13327	23810.80688	15534.17471
dUTP	NA	NA	NA	NA	7765.619081	8732.649151	NA	NA	NA	NA	NA	NA
ethanolamine	173751.5021	116361.8939	165002.3497	112091.8176	144449.6085	95515.00936	62975.61868	128543.4963	141522.8054	137813.2896	80760.18683	94572.54718
FAD	2445690.984	1474722.775	1453113.46	1038202.029	2014778.694	1435408.479	833391.2989	1705174.529	1495490.325	1029514.641	882884.608	1006437.956
flavone	NA	53079.40077	15474.47077	28841.28612	NA	9140.615864	24780.36638	39753.97093	42499.11696	45205.98268	15737.42721	9286.735733
FMN	NA	NA	NA	NA	NA	NA	NA	NA	NA	NA	NA	NA
folate	159482.9164	138738.0437	129937.0665	59583.06185	128342.4242	62567.18928	52335.38386	126304.8068	187983.8378	140741.7896	26921.88662	26913.70826
fructose-1,6-bisphosphate	19341991.92	14100264.21	6056011.482	10085211.18	9003581.745	4530715.371	4071491.554	8893754.32	13442125.24	8898602.561	2559315.375	981343.4061
fructose-6-phosphate	620108.5023	505235.3498	194573.9129	175041.5621	236086.1227	206615.1257	132395.0824	223069.6483	367602.9102	250850.3217	143282.5332	88048.94543
fumarate	13485312.21	8591191.202	9016540.34	7591507.532	9423799.847	7509410.262	10483732.47	8823219.034	6912344.891	5950286.638	8558338.032	9972413.871
GDP	2707547.805	1123722.431	714967.6433	465401.9617	856571.7206	549213.2578	976036.9785	1241908.77	974518.5917	485947.5653	435291.0782	339626.616
geranyl-PP	111900.3046	89328.63168	89685.498	31046.4598	53108.9741	37926.40561	72708.19926	61592.52686	NA	NA	36117.08151	21075.10193
glucono-1,5-lactone	107674.9445	87945.32501	77317.33052	41375.46107	82813.60219	42521.88975	46836.59201	66812.32421	61234.4716	43581.40115	28929.37038	27438.24375
glucosamine	274632.1957	286039.2305	198262.6418	172649.8373	65833.38654	87294.34392	310144.1409	356931.6817	270768.6552	222379.6801	190670.5346	103516.4996
glucose-1-phosphate	777645.9579	853291.6814	569992.0675	274315.9559	456644.6816	382857.275	474699.1661	694127.287	635628.0476	390557.897	228073.3887	248096.7342

glucose-6-phosphate	428377.1105	396757.1877	198504.5417	172962.9789	213168.7048	242868.4927	259010.1132	212429.339	255809.7135	195504.7244	178327.1876	92225.45841
glutamate	145479959.6	119843932.5	121417454.4	107853420.9	147661072.4	109763697.1	99840394.76	102754676.7	146533547	127166957.7	99503694.63	86147969.56
glutamine	103073808.5	113129792	118941993.6	97349048.91	117299716.1	110158377.4	101455132.4	106414846.3	121000449.9	109478375.4	96435705.07	84264135.48
glutathione	45558078.7	16706122.75	20113455.14	5389696.917	20934367.92	21802500.43	32738095.96	26639819.58	23756247.1	7347215.803	21202696.71	22056135.36
glutathione disulfide	13895845.94	24673402.01	20809087.25	20193380.02	24273354.67	15665246.81	12344007.07	20240636.44	16951430.68	18122387.04	11447566.24	7705343.069
glycerate	293174.5457	330939.3347	294058.972	641422.2596	492031.8324	238225.3807	65492.42844	247115.25	374429.0165	413165.4335	198235.5565	142631.2518
glycerophosphocholine	180810678.7	140741585.4	154815742.1	132554202.3	171383462.7	140714877.6	111354250.4	133470211.1	182445569.9	135367961.1	111217866.4	80231832.13
glycine	8282.689238	8281.87933	7251.355565	NA	7242.671378	5176.196759	6718.913273	NA	NA	NA	NA	NA
glycolate	37412.1762	27217.59657	19669.72313	17456.02506	35321.70308	32973.28587	19002.37842	23018.87754	5430.14579	NA	27223.35804	16794.87801
glyoxylate	53702.40283	37600.91839	31788.52372	21418.53625	36121.66329	32529.7327	41828.72818	48119.60945	40041.56001	28388.96857	42564.45528	50721.26774
GMP	16685327	18409049.5	22153143.73	22999501.32	23373208.04	25590254.05	20012657.06	21909826.86	18862909.83	19790333.09	24006356.21	22666518.62
GTP	2393381.027	1070308.38	707237.3268	557285.1253	1011681.306	724817.6646	896370.9014	762177.3196	1478529.578	509624.4286	531147.5203	236468.3883
guanidoacetic acid	607931.3177	274594.6716	228348.1047	205481.4401	472270.7698	188588.4174	226081.9681	234891.8637	288918.529	187535.4732	142165.0506	213846.1538
guanine	206196.3021	182941.7224	194635.4123	81418.24234	206346.2399	89309.62732	89823.94515	149124.2798	184639.0436	66464.6873	99205.08492	47670.92446
guanosine	291838.5815	287863.0557	390277.1135	189406.1235	302548.344	193485.2721	315275.4223	241849.4932	365403.2571	363484.8174	260428.2685	137154.8892
guanosine 5-diphosphate,3-diphosphate	70252.75629	55369.37742	31056.27985	13977.68658	14493.28147	27962.14604	11906.25267	36751.4297	44002.39545	23297.28877	12960.14748	NA
hexose-phosphate	29646718.45	19840305.48	16464051.68	9489478.945	18938813.92	13289088.12	19350889.7	14638600.84	18717693.93	14100934.1	10391926.77	12150851.14
histidine	9384910.491	13102810.44	14712356.3	7940205.232	10034936.71	12849885.4	6515478.683	9968530.758	9469726.55	8712948.423	9520849.152	5943528.312
histidinol	167166.1495	57079.83072	71967.11194	61086.26122	117013.0493	63679.32302	40851.58365	56941.56403	78168.28822	54866.77798	52280.95111	23296.46262
homocysteic acid	149654.5989	111756.2942	70408.50955	33051.13904	186599.3834	66229.56146	48424.71937	123351.1318	38847.33431	21222.83685	50762.00958	111746.7106
homocysteine	146675.0894	87815.20472	130153.2854	99248.72815	172915.1439	86584.37055	132257.4677	72265.06673	228022.6505	144432.4506	122576.2576	58128.50535
homoserine	95385.06348	88946.9651	78585.53504	70594.59537	81475.87421	77129.99464	74783.71967	99294.41452	118711.2778	102385.7731	62914.90731	37350.59185
hydroxyisocaproic acid	3117452.148	3188737.913	2269863.536	1135002.754	2285467.307	1408908.241	1682529.779	1380428.98	1293737.957	1920137.941	1172888.161	937464.2153
hydroxyphenylacetic acid	220657.2197	318239.6187	189370.8571	69735.89068	182287.1455	38875.55908	76578.24288	157564.8996	89883.79604	113389.9465	49958.92369	44640.98523
hydroxyphenylpyruvate	12886.81806	NA	NA	NA	NA	NA	NA	NA	NA	NA	NA	NA
hydroxyproline	591444.2363	544654.3766	490661.4148	477646.9215	579691.3732	430547.3495	406537.033	481649.1426	617302.1467	452274.3861	401656.5309	361607.2297
hypoxanthine	20750.79414	14428.72021	41333.13629	40981.10022	NA	9289.01718	25382.76229	NA	21160.67073	20320.61	18852.01471	33285.13455
IDP	2898501.129	2137937.364	1684640.964	1209860.261	2008871.709	1653699.077	1591695.249	1779204.225	1424103.44	913266.1887	1187580.354	959222.9249
imidazole	NA	NA	8461.894394	7544.510597	NA	NA	17848.12374	NA	11241.6957	14151.39716	16620.88981	16810.98862
imidazoleacetic acid	101702.4593	56189.87223	50453.91549	28808.74819	37870.79375	60496.59186	37451.26109	80384.67088	64203.02365	51214.36245	61178.88161	25051.67835
IMP	29359143.77	26783343.08	25452071.19	21612157.85	31035330.01	20742333.61	23057897.28	26097432.5	24095474.59	23657417.46	20526706.9	19810281.91
indole	359874.4528	158015.9284	169643.8693	88052.9575	248387.0955	148403.3583	120630.5918	164182.9322	209085.3434	174729.0211	66255.99961	93342.63236
indole-3-carboxylic acid	248237.6687	118821.035	101092.8941	78095.82365	127523.5643	92935.57343	150716.8981	122883.2325	78174.31485	72741.11348	50892.25744	148127.1337
indoleacrylic acid	85109.07839	106329.9146	50873.6171	113973.8695	142906.5528	65620.63277	50214.25145	100104.0149	61876.91932	56304.42787	36452.4605	38841.43852
inosine	77564317.14	77702575.5	77559034.5	62616100.81	77413459.26	72358047.82	79151759.44	77020268.31	70911097.78	75824269.62	73883253.27	68602630.89
isocitrate	98115.43609	45863.15703	36985.81536	28200.84504	43330.21759	27890.02747	46589.11269	56542.46287	66316.81543	25276.34739	41870.88697	40623.70848
kynurenic acid	907190.0999	668826.0956	383672.2969	279337.2071	450477.2707	337927.1123	531430.2699	658312.2178	320923.8	245408.573	454041.7992	491595.1783
kynurenine	24434.71388	22702.38812	12457.8367	12532.09604	36506.16483	18109.28496	NA	17238.82809	30713.89033	18216.16627	12461.83524	13347.66974
lactate	177315439.5	126379665.1	117780275.3	103086802.1	141948585	104687363.7	106421653.4	116024627.7	118235343.3	94021827.87	106042267.3	108058931.4
L-arginino-succinate	419506.8382	390490.0316	363048.4464	478038.8974	481344.699	389099.2881	336787.0227	357936.0134	450893.6743	305340.7276	347280.2627	457940.3993
leucine-isoleucine	40501830.73	26058856.73	21020604.6	15303733.42	28334905.78	17868803.61	15218400.91	23037647.84	27407207.2	27436263.6	12253855.96	11950748.36
lipoate	NA	NA	NA	NA	NA	NA	NA	NA	NA	NA	NA	NA
lysine	7631270.849	4111944.591	2991335.436	2133661.107	5571956.573	2562350.791	1505525.303	2516277.74	6665493.043	3986856.052	1059482.018	1208954.326
malate	25949725.27	17305619.84	17991729.44	23432132.75	23962505.4	14720433.79	27054514.9	26104087.61	15999084.57	10995504.98	26107886.78	35042218.28
maleic acid	14006456.25	8631220.317	8689053.537	7236480.384	10178206.39	7810726.077	8915056.73	8785812.775	6848590.103	5452948.108	8327901.728	9254144.017

malonyl-CoA	NA	NA	NA	NA	NA	NA	NA	NA	NA	NA	NA	NA
methionine	1575771.292	1387939.976	1361411.296	945568.5106	1647507.669	1117624.652	986286.3207	1376471.896	1798716.702	1365385.818	928239.9229	712320.2147
methionine sulfoxide	172282.929	145468.0599	195693.1629	114922.0855	146150.0294	114874.1479	104805.0831	133632.2411	189286.2482	88010.01195	71404.19939	61624.43929
methylcysteine	595778.8613	470863.5934	377559.7197	257016.3017	402429.0185	348335.1682	323450.2877	366231.5581	459038.2561	333600.9056	318559.2331	296839.0517
methylmalonic acid	19690948.11	11941226.84	9214731.114	10861839.22	10951995.42	9423046.018	14019237.44	12785296.18	11674859.86	10270040.05	11513315.4	8312987.581
methylnicotinamide	NA	NA	NA	NA	NA	NA	NA	NA	185185.56	148796.9246	NA	NA
myo-inositol	130251649.3	109611774.5	101438261.6	78784856	120074111.8	85461275.95	79026273.67	97393523.12	107701031.5	89047498.17	56689112.49	56543347.05
N6-Acetyl-L-lysine	44754275	21206095.45	19861194.09	9934751.782	24536941.74	14182182.36	8202271.598	12783752.63	36792224.5	11010209.68	5307265.648	5190031.264
N-acetyl-glucosamine	74632.07618	51126.6145	45153.40416	38163.30612	74019.15057	41998.50521	49195.34717	61982.06898	59421.64849	44848.09908	42296.39916	52127.35379
N-acetyl-glucosamine-1-phosphate	998345.0919	884856.8263	819846.7236	422435.5527	841575.982	512348.3414	587970.9617	706151.8372	637991.9384	579443.5668	466930.5513	408280.3589
N-acetyl-glutamate	1552105.448	744729.5194	685136.6892	513898.8704	987217.5728	543055.7417	473714.0774	773912.0814	1020248.525	682617.2906	434230.9028	418785.1921
N-acetyl-glutamine	1770375.394	890840.5877	813346.8925	210855.9656	1490995.632	230280.5224	483176.6373	738496.563	1253329.587	827015.8461	304844.4527	425735.766
N-Acetyl-L-alanine	141267136.6	109721120.5	101786852.1	88440432.68	113179608.7	83704678.64	91156643.06	96451074.15	95719914.13	87196070.62	74163334.14	76833546.53
N-acetyl-L-ornithine	64411.32261	32176.83993	36741.81303	32704.84985	70556.95358	41680.0814	41996.24977	44188.37166	71423.85295	37816.96499	29215.3968	25898.58587
N-Acetylputrescine	NA	NA	NA	NA	NA	NA	NA	NA	NA	NA	NA	NA
NAD+	16877569.68	9332142.668	10487125.08	5813318.714	11306579.07	6263784.586	12631222.37	11269894.11	11065887.23	6412327.201	12618039.39	3449276.518
NADH	983353.8952	1338905.007	1181271.053	373876.9473	832140.6516	2390691.505	272833.7006	685713.5479	992333.5145	608674.2964	820517.8248	137199.8495
NADP+	759336.3819	826650.0255	869832.7003	542647.0621	718688.4527	767118.5463	994443.5265	978800.0266	751097.265	667378.7495	762405.8308	280453.0766
NADPH	NA	NA	NA	NA	NA	10869.54727	NA	NA	NA	NA	NA	NA
N-carbamoyl-L-aspartate	447761.3484	265768.7048	254227.5202	321517.0311	357573.149	184304.604	176532.8016	188010.8429	375839.2284	240638.8291	234012.8083	135674.3436
Ng,NG-dimethyl-L-arginine	2011683.659	1450489.711	660060.2879	753419.4359	1049849.998	721969.3816	650915.7188	857520.4796	2552835.972	742927.9645	623788.0583	537260.227
nicotinamide	265647130.5	180660871	231556219.9	191480624.8	306451334	185405155.9	133873415.8	187505964.5	220541794.3	217004979.1	73390944.99	132336700.3
nicotinamide ribotide	373527.3511	428737.0336	360564.7451	280952.8724	487448.9998	315865.1117	308783.1745	456432.6854	400599.4542	283329.1888	267214.7419	204727.3851
nicotinate	80658.25031	110715.7709	91820.70602	100911.1884	114051.9674	91807.62122	289529.4402	178831.1693	292295.7233	181124.5896	187919.8764	134113.0292
O8P-O1P	965428.2784	598096.4945	588845.5062	522057.233	692001.1253	452335.3005	509156.2319	572073.8853	603652.9326	457189.4613	404474.9999	375356.183
O-acetyl-L-serine	62876.01674	54666.34083	23409.51547	21129.4519	52580.71136	29811.08273	21517.91121	34425.55748	47293.77563	29954.39987	12662.07936	16913.5304
OBP	223966.9668	210777.6995	232870.9806	133872.4902	168945.2715	140922.4895	174240.4587	155188.4376	145542.779	117393.517	117490.0377	133067.2881
ornithine	80933.96604	91498.7868	58520.43512	67292.77061	125420.6396	75196.72068	45109.37488	74098.38257	74818.37558	66330.53699	49880.27052	39817.02957
orotate	259097.1495	160412.1072	156092.6827	125140.5913	135328.3577	118706.42	245755.4159	206210.6184	147318.1003	111221.0403	98134.50333	164581.0905
orotidine-5-phosphate	NA	NA	NA	NA	NA	9318.981495	NA	9318.434492	NA	NA	9730.485761	7761.059797
oxaloacetate	323250.4449	188766.8441	143434.2362	192945.5319	250995.2877	259128.4563	282183.7832	141374.671	196391.3442	118644.945	250563.1327	397288.6883
p-aminobenzoate	1035397.022	660397.1002	422954.7475	359890.9693	669780.4757	263716.311	462880.9957	452339.7739	919830.5679	805311.9212	342515.6392	171022.3976
pantothenate	11936726.41	5972719.589	5867332.318	3975279.965	7262899.018	4440083.436	4537625.252	4989405.424	4784085.495	2995888.908	4240818.286	4313290.881
phenylalanine	18105472.59	8355788.926	8831016.735	6037239.088	12541670.84	7694026.555	5728747.218	7591297.501	14520527.92	8964875.07	4176552.483	4423994.709
phenyllactic acid	443236.2187	433735.9605	271608.466	308548.6583	402053.2159	145471.5739	197248.6208	203078.9638	247902.3877	276419.7842	121148.948	184818.7432
phenylpropiolic acid	465390.7013	159204.0834	151651.1799	121199.0189	284205.7514	185763.3249	382015.3103	299063.5244	245811.3641	157218.1414	195372.0865	349384.1204
phenylpyruvate	13964.95086	19664.23534	13980.3043	NA	24884.31281	NA	NA	9835.835638	12433.78094	7250.3268	NA	NA
phosphoenolpyruvate	2519964.053	504950.1852	210692.099	48533.91831	202708.5615	87929.69788	637592.7375	197451.9616	739830.3471	106831.1765	248503.8765	95300.41173
phosphorylcholine	246130515.6	204912098.4	197673993.9	147475914.1	220587929.5	183546892.5	161285739	183168011.2	232738253.8	197931553.5	112989396.3	101442807.4
p-hydroxybenzoate	2699343.004	2594169.588	3596813.529	660560.194	2236472.362	850056.9498	1212041.175	1558219.045	1729371.788	1257507.444	1106945.075	510630.8633
prephenate	NA	NA	NA	NA	NA	NA	NA	NA	NA	NA	NA	NA
proline	28600135.22	25511682.19	27616227.81	18664176.1	31008693.3	19568749.92	14106502.53	27318227.83	25970931.11	28371650.15	14189891.43	10386350.74
propionyl-CoA	77820.76954	20629.13401	33518.76487	19161.59049	51735.3012	37794.85822	25360.03035	21206.96513	40901.01454	14493.11797	25369.56734	26911.31096
purine	341184.3026	185727.8286	155704.3111	102921.864	278363.2022	132042.9085	89262.84967	160159.1841	279512.2405	131688.0991	74573.26594	71720.52309
putrescine	63250.97624	35561.69984	24256.76529	111678.1938	10334.12589	26096.89144	156410.5229	NA	4424.910408	89168.32638	20000.78516	43522.15452

pyridoxamine	22923.40624	NA	30612.09366	42509.31361	25249.62063	22440.46581	17943.53041	NA	106276.7618	NA	NA	NA
pyridoxine	10612.47838	NA	8137.035531	NA	11999.81702	NA	14710.16898	NA	NA	11623.65549	NA	NA
pyroglutamic acid	293286.9488	337365.432	438658.5364	382043.3547	423803.5081	240101.1933	313024.2548	266871.0523	565241.4339	433385.6962	238814.3676	304103.5715
pyrophosphate	7036140.187	3756845.743	3766091.548	2212201.942	4411417.609	2343562.305	2830140.615	3217884.365	3731159.742	2716701.647	1758716.871	1814230.867
pyruvate	121034.6931	81168.68413	69860.70736	50165.28604	63502.1156	50632.2085	67685.26445	74132.16627	59626.78951	42370.12315	71821.38749	56917.2134
quinolinate	6454.44333	8937.973048	10613.80095	20848.37616	13297.46891	13074.73195	6689.957007	NA	51666.66337	6551.53277	NA	17526.21617
riboflavin	128130.1646	174685.4209	128562.1013	107103.3679	220021.04	183810.1034	82681.39115	169221.0961	97328.16214	130098.0203	88018.24446	98497.95733
ribose-phosphate	6075940.35	6279360.354	8815013.922	4853857.363	9816551.351	4786864.026	6237090.962	6120760.667	8498418.26	8103977.457	3085741.034	5174364.541
S-adenosyl-L-homocysteine	1224109.391	1002768.269	964745.0387	273048.275	1012267.447	552039.4621	588320.8522	743330.1328	461462.5828	407066.6607	662605.5753	431061.9383
S-adenosyl-L-methioninamine	NA	NA	NA	NA	NA	NA	NA	NA	NA	NA	NA	NA
S-adenosyl-L-methionine	12590355.7	6909355.425	6161508.114	4013622.162	8019497.269	4618799.713	2851948.821	5468207.914	12366113.4	6203951.433	2638436.898	2539546.565
sarcosine	13844474.64	12121490.62	11095540.68	9847736.354	11359222.94	8623944.531	8757693.127	11138260.3	12712959.81	13279543.34	7230927.308	5857793.102
SBP	1432807.794	1144937.245	955518.0198	499451.5926	977207.1653	754133.1792	670325.7665	600907.9332	838569.4908	746321.6721	383308.9361	360632.4124
serine	14895640.64	16405053.41	13955887.88	15133823.75	16579571.32	14595576.49	12015885.3	14537984.73	15242901.84	11772528.78	9990076.17	10139727.78
shikimate	1715475.767	1217469.694	86602.43625	84387.7385	137187.1518	167734.166	122181.0481	665235.9352	101449.303	363163.9169	23343.81481	10859.65139
shikimate-3-phosphate	14893.98155	NA	13481.87366	NA	13442.34214	NA	4749.266464	30664.63476	24911.77904	NA	NA	NA
S-methyl-5-thioadenosine	NA	7572009.685	3882910.123	3375131.968	3302427.947	1175398.443	2926155.546	5220731.263	8762036.323	4231912.836	3572021.918	1789056.962
sn-glycerol-3-phosphate	22082392.07	15311268.05	10790382.72	5376478.241	12395981.77	2921966.159	5271758.992	10408798.88	9518761.001	8916785.518	11031480.84	854685.9068
spermidine	189124.0971	257175.2412	225660.9438	322789.7853	152046.6756	259112.9264	402632.3977	222216.904	147883.4679	200002.3902	245220.1273	335050.5461
spermine	NA	NA	NA	NA	NA	NA	NA	NA	NA	NA	NA	NA
S-ribosyl-L-homocysteine	27903.38571	16637.47297	32606.77834	16060.26041	39823.0504	28482.16792	27956.35651	29508.08502	42451.10166	12940.07942	14994.46921	21747.07027
succinate	17599315.14	12828605.06	7647918.013	9009928.922	8523450.124	5689288.652	11476707.44	9788575.31	9725936.298	8492821.482	8868729.824	6537057.064
succinyl-CoA-methylmalonyl-CoA	NA	NA	NA	NA	NA	NA	NA	NA	NA	NA	NA	NA
succinyl-CoA	94361.54969	18488.69509	30019.07416	7248.469659	22809.99099	22778.9917	40226.34557	12424.62831	16604.48719	9271.204484	7768.980353	13458.68414
taurine	162427803.3	145632470.9	141457786.3	114083513.5	166896456.5	116809694.4	115596284.8	135862410.5	153201323.8	135031512.7	107059595.3	93284731.93
taurodeoxycholic acid	12941.55234	9866.533319	7758.750092	NA	8801.456493	NA	NA	7765.271757	NA	NA	NA	NA
thiamine	NA	NA	NA	NA	NA	NA	NA	NA	NA	NA	NA	NA
thiamine pyrophosphate	926126.9714	535934.3061	325760.644	219896.0854	361337.6341	284756.0036	440892.8241	539976.5298	298993.3199	215760.9076	314656.8151	145260.4857
thiamine-phosphate	345436.6807	290662.3921	285781.2423	275296.4542	408709.6949	292090.0621	71344.19156	263948.8772	498369.9868	332372.8878	146804.7239	159191.653
threonine	21015836.26	16097775.27	18679167.63	12007322.83	20019022.66	12390866.12	10787813.76	15297094.94	21813401.06	17658679.76	6663398.488	8079931.757
thymidine	NA	38873.39959	64709.73434	7638.893134	26044.23492	17764.0311	29365.3951	41752.64093	69372.06006	34685.82259	21745.37609	40244.27617
thymine	18205.03296	11237.82278	12298.54415	19559.01424	19729.1311	35632.24578	43011.8996	24632.3078	20973.58055	32858.51043	18422.29064	16443.98808
trans, trans-farnesyl diphosphate	12442.31088	12697.56811	9174.374704	NA	20978.44953	NA	NA	9707.042578	16020.36101	NA	NA	NA
trehalose-6-phosphate	64797.02911	131114.6185	130447.9112	83051.2155	32676.7791	93374.92323	70923.07646	124235.4007	130908.7524	89403.48347	10938.94645	47347.18248
trehalose-sucrose	597766.3522	771533.46	686798.9033	558707.7891	2097766.871	840508.5563	1419655.168	1625022.292	1088979.252	216619.9723	268835.5185	2008655.714
tryptophan	2722119.873	1475489.923	1408215.204	1072303.231	2345240.065	1417964.318	1053116.448	1514671.528	2397541.369	1353471.523	839000.8189	950544.1569
tyrosine	1013123.542	790231.2231	948897.3892	624376.3179	831417.0279	789439.419	685315.2923	803409.1569	1095975.02	766591.7152	625287.3759	686332.1108
UDP-D-glucose	7362551.85	3632674.954	3316276.041	2281531.353	4772625.558	2365731.543	2469390.179	3334379.78	3056495.785	1962131.813	1885061.59	2080848.763
UDP-D-glucuronate	351138.1146	168762.4965	227262.8892	94741.59403	261954.1609	163074.5169	115438.9778	164629.274	164528.8901	141333.4242	136158.3377	99919.70154
UDP-N-acetyl-glucosamine	1663283.789	803399.0257	974793.6644	555004.7035	1143922.486	625768.826	491340.3442	809447.7362	783695.4334	572685.3251	492301.3918	418839.5168
UDP	1696884.859	1587233.108	1230595.147	768903.7861	1518424.565	1268098.03	795577.4508	1454109.147	769470.841	636266.2431	843838.2922	571842.1533
UMP	1159293.096	1136207.782	1213479.626	1003553.094	1387025.87	1184937.934	626764.6361	1224447.727	1273624.886	914688.984	919151.6625	930629.7982
uracil	14256055.49	11137976.37	11163447.83	3713587.634	13428429.98	4575515.19	9452766.198	8342287.983	7184214.685	7993778.119	5832209.591	4159175.559
urea	14824828.98	10438777.62	8541177.676	5471974.606	12056776.73	6027389.758	4773763.285	7330492.787	12616065.16	9014821.901	5527767.059	3734722.383
uric acid	289592.8205	111902.9181	126328.4632	52952.79104	116063.5672	62449.62829	102408.6012	113330.2892	112171.1574	45984.33595	74465.03162	167224.163

uridine	32766.28059	NA	NA	NA	21617.22258	11549.71481	NA	19769.56043	13509.75182	NA	NA	NA
UTP	516256.3514	614999.4929	447506.4656	103569.0844	461640.8792	465056.9595	137067.222	335982.2949	362115.2533	129918.6893	270715.9204	129423.4464
valine	538594.3087	662106.6196	525266.397	367335.9719	674816.3953	467973.4063	371986.2016	457095.897	554086.6996	642428.8598	274784.2618	306924.7511
xanthine	8924803.917	5590722.778	6753322.519	2362644.548	5307699.027	3127835.33	6124557.157	4089704.487	4444027.983	3579519.202	3111547.876	3430253.652
xanthosine	164134.9488	484523.1471	137437.9557	67902.21977	444139.7048	75963.98836	161359.5875	72267.69942	73926.60833	87325.23421	100528.5434	79378.72209
xanthosine-5-phosphate	NA	13534.89736	NA	NA	8293.100653	7739.189104	NA	NA	NA	NA	NA	NA
xanthurenic acid	202521.232	159095.6727	123392.2452	114563.8774	130014.2811	113126.1271	234666.2896	169749.9245	87429.02911	64301.81176	147296.9387	132954.0227

NA: not available.
